# Supplementary material for: A public vaccine-induced human antibody protects against SARS-CoV-2 and emerging variants
Source: bioRxiv. 2021 Mar 24:2021.03.24.436864. Preprint. [Version 1] doi: 10.1101/2021.03.24.436864 (PMC8010723; doi:10.1101/2021.03.24.436864)
Supplement: 1 [file NIHPP2021.03.24.436864-supplement-1.pdf]

bioRxiv preprint doi: https://doi.org/10.1101/2021.03.24.436864; this version posted March 25, 2021. The copyright holder for this preprint (which was not certified by peer review) is the author/funder, who has granted bioRxiv a license to display the preprint in perpetuity. It is made available under aCC-BY 4.0 International license.

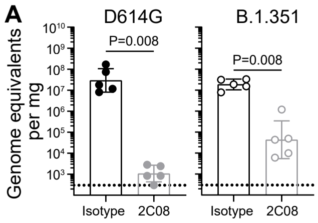

Figure S1. mAb 2C08 protects hamsters from SARS-CoV-2 challenge. (**A**) Lung viral RNA titer using 5' UTR probe of hamsters that received isotype (black) or 2C08 (grey) one day prior to intranasal challenge with 10^5 $TCID_{50}$ D614G (*left*) or B.1.351 (*right*) SARS-CoV-2 variants. Bars indicate geometric mean ± geometric SD, and each symbol represents one hamster. Data are from one experiment, n = 5 per condition. *P*-values from two-tailed Mann-Whitney tests.

bioRxiv preprint doi: https://doi.org/10.1101/2021.03.24.436864; this version posted March 25, 2021. The copyright holder for this preprint (which was not certified by peer review) is the author/funder, who has granted bioRxiv a license to display the preprint in perpetuity. It is made available under aCC-BY 4.0 International license.

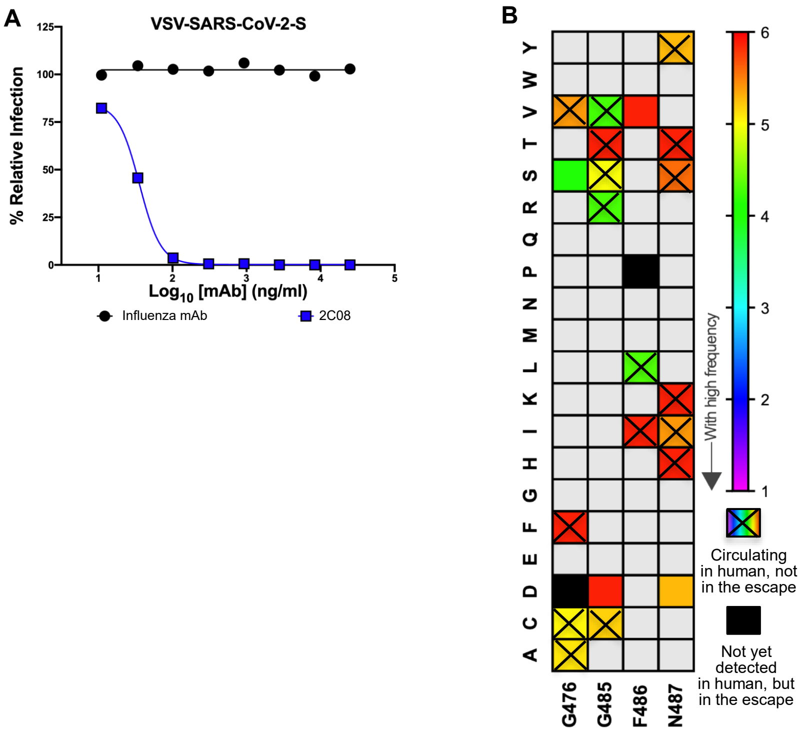

Figure S2. Escape mutant mapping of mAb 2C08. (**A**) 2C08 and a control anti-influenza virus mAb were tested for neutralizing activity against VSV-SARS-CoV-2. The concentration of 2C08 added in the overlay completely inhibited viral infection. Data are representative of two independent experiments. (**B**) 2C08 escape profile in currently circulating SARS-CoV-2 viruses isolated from humans. For each site of escape, we counted the sequences in GISAID with an amino acid change (829,521 total sequences at the time of the analysis). Variant circulating frequency is represented as a rainbow color map from red (less circulating with low frequency) to violet (most circulating with high frequency). A black cell indicates the variant has not yet been isolated from a patient. A rainbow cell with cross indicates the variant has been isolated from a patient, but not appear in those 2C08 mAb escape mutants.

bioRxiv preprint doi: https://doi.org/10.1101/2021.03.24.436864; this version posted March 25, 2021. The copyright holder for this preprint (which was not certified by peer review) is the author/funder, who has granted bioRxiv a license to display the preprint in perpetuity. It is made available under aCC-BY 4.0 International license.

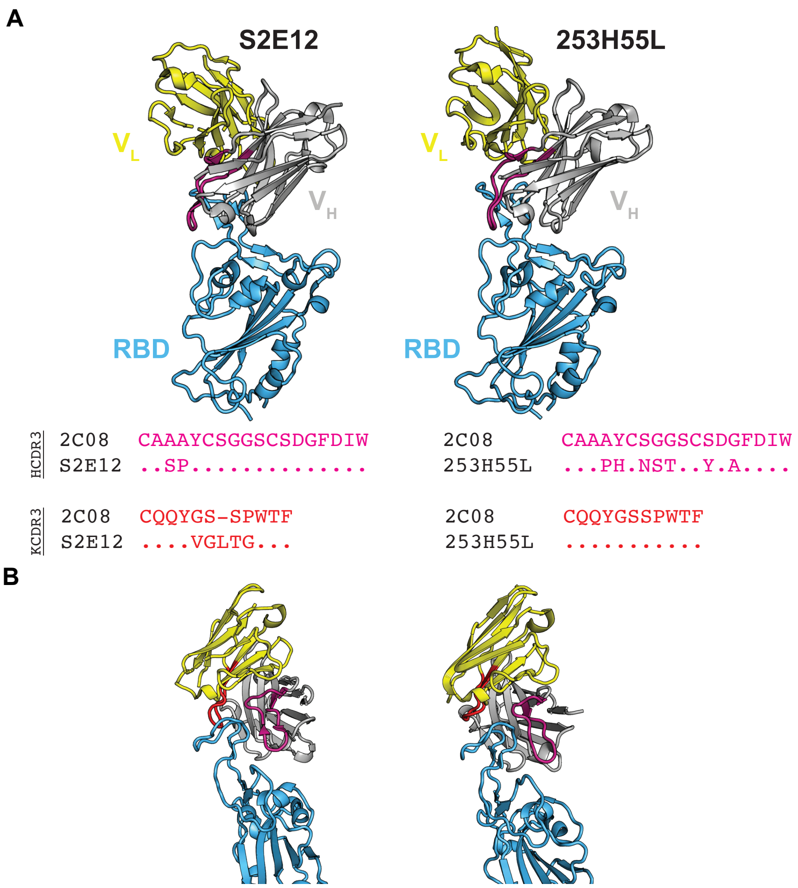

Figure S3. mAb 2C08 recognizes a public epitope in SARS-CoV-2 RBD. (**A and B**) Structures of mAbs S2E12 (PDB 7K45) and 253H55L (PDB 7ND9) complexed with RBD and their heavy (pink) and light (red) chain CDR3 sequence alignments with 2C08.

Table S1.

mAb	Induced after SARS-CoV-2	Publication	Heavy Chain V-GENE and allele	Heavy Chain J-GENE and allele	Heavy Chain D-GENE and allele	Heavy Chain HCDR-IMGT lengths	Heavy Chain HCDR3	Light Chain V-GENE and allele	Light Chain J-GENE and allele	Light Chain LCDR-IMGT lengths	Light Chain LCDR3
2C08◊	mRNA vaccine		IGHV1-58*01	IGHJ3*02	IGHD2-15*01	8.8.16	AAAYCSGGSCSDGFDI	IGKV3-20*01	IGKJ1*01	7.3.9	QQYGSSPWT
S2E12◊	Infection	(*25*)Tortorici et al., 2020	IGHV1-58*01	IGHJ3*02	IGHD2-15*01	8.8.16	AAPDCNRTTCRDGFDI	IGKV3-20	IGKJ1	7.3.9	QQYGSSPWT
COVD57_P2_H6^	Infection	(*24*)Robbiani et al., 2020	IGHV1-58*02	IGHJ3*02	IGHD2-15*01	8.8.16	AAPYCSGGSCNDAFDI	IGKV3-20*01	IGKJ1*01	7.3.9	QQYGSSPWT
COV107_P2_81^	Infection	(*24*)	IGHV1-58*01	IGHJ3*02	IGHD2-15*01	8.8.16	AAPYCSGGSCSDAFDI	IGKV3-20*01	IGKJ1*01	7.3.9	QQYGSSPWT
MOD8.7.P1_C7	mRNA vaccine	(*15*)Wang et al., 2021	IGHV1-58*01	IGHJ3*02	IGHD2-15*01	8.8.16	AAPYCSGGSCYDAFDI	IGKV3-20*01	IGKJ1*01	7.3.9	QQYGSSPWT
MOD8.7.P1_E3	mRNA vaccine	(*15*)	IGHV1-58*01	IGHJ3*02	IGHD2-15*01	8.8.16	AAPYCSGGSCYDAFDI	IGKV3-20*01	IGKJ1*01	7.3.9	QQYGSSPWT
MOD8.7.P1_F5	mRNA vaccine	(*15*)	IGHV1-58*01	IGHJ3*02	IGHD2-15*01	8.8.16	AAPYCSGGSCYDAFDI	IGKV3-20*01	IGKJ1*01	7.3.9	QQYGSSPWT
COV2-2196◊	Infection	(*23*)Zost et al., 2020	IGHV1-58*01	IGHJ3*02	IGHD2-2*01	8.8.16	AAPYCSSISCNDGFDI	IGKV3-20*01	IGKJ1*01	7.3.10	QHYGSSRGWT
COVD21_P2_F9^	Infection	(*24*)	IGHV1-58*01	IGHJ3*02	IGHD2-15*01	8.8.16	AAPHCSGGSCLDAFDI	IGKV3-20*01	IGKJ1*01	7.3.9	QQYGSSPWT
COVD21_P1_F10	Infection	(*24*)	IGHV1-58*01	IGHJ3*02	IGHD2-15*01	8.8.16	AAPHCSGGSCYDAFDI	IGKV3-20*01	IGKJ1*01	7.3.9	QQYGSSPWT
MnC5t2p1_G1^	Infection	(*26*)Kreer et al., 2020	IGHV1-58*01	IGHJ3*02	IGHD2-15*01	8.8.16	AAPRCSGGSCYDGFDI	IGKV3-20*01	IGKJ1*01	7.3.9	QQYGSSPWT
COVD57_P1_E6	Infection	(*24*)	IGHV1-58*02	IGHJ3*02	IGHD2-15*01	8.8.16	AANHCSGGSCYDGFDI	IGKV3-20*01	IGKJ1*01	7.3.9	QQYGSSPWM
HbnC3t1p1_C6^	Infection	(*26*)	IGHV1-58*01	IGHJ3*02	IGHD2-2*01	8.8.16	AAPHCSSTICYDGFDI	IGKV3-20*01	IGKJ1*01	7.3.9	QQYGSSPWT
MOD3.73.P2_B6	mRNA vaccine	(*15*)	IGHV1-58*01	IGHJ3*02	IGHD2-8*01	8.8.16	AAPYCSNGVCHDGFDI	IGKV3-20*01	IGKJ1*01	7.3.9	QQYGSSPWT
COV2-2381◊	Infection	(*23*)	IGHV1-58*01	IGHJ3*02	IGHD2-2*01	8.8.16	AAPYCSRTSCHDAFDI	IGKV3-20*01	IGKJ1*01	7.3.10	QHFGSSSQWT
MOD11.59.P1_D1	mRNA vaccine	(*15*)	IGHV1-58*01	IGHJ3*02	IGHD2-2*01	8.8.16	AAPYCSSTSCHDGFDI	IGKV3-20*01	IGKJ1*01	7.3.9	QQYGSSPWT
HbnC3t1p2_C6^	Infection	(*26*)	IGHV1-58*01	IGHJ3*02	IGHD2-2*01	8.8.16	AAPYCSSTRCYDAFDI	IGKV3-20*01	IGKJ1*01	7.3.9	QQYGRSPWT
COV107_P1_53	Infection	(*24*)	IGHV1-58*01	IGHJ3*02	IGHD2-2*01	8.8.16	AAPHCSSTSCFDAFDI	IGKV3-20*01	IGKJ1*01	7.3.9	QQYGNSPWT
COV2-2072◊	Infection	(*23*)	IGHV1-58*02	IGHJ3*01	IGHD2-2*01	8.8.16	AAPHCNRTSCYDAFDL	IGKV3-20*01	IGKJ1*01	7.3.9	QQYGSSPWT
COV072_P3_42	Infection	(*24*)	IGHV1-58*01	IGHJ3*02	IGHD2-2*01	8.8.16	AAVDCNSTSCYDAFDI	IGKV3-20*01	IGKJ1*01	7.3.9	QQYDISPWT
C004.8.P1_G10^	mRNA vaccine	(*15*)	IGHV1-58*01	IGHJ3*02	IGHD2-2*01	8.8.16	AAPHCNRTSCFDGFDI	IGKV3-20*01	IGKJ1*01	7.3.9	QQYGSSPWT
C004.8.P2_E3	mRNA vaccine	(*15*)	IGHV1-58*01	IGHJ3*02	IGHD2-2*01	8.8.16	AAPDCNRTTCRDGFDI	IGKV3-20*01	IGKJ1*01	7.3.9	QQYGSSPWT
MOD6.24.P2_A7*	mRNA vaccine	(*15*)	IGHV1-58*01	IGHJ3*02	IGHD2-2*01	8.8.15	AAVYCTTTCSDAFDI	IGKV3-20*01	IGKJ1*01	7.3.9	QQYDISPWT
mAb55*^	Infection	(*42*)Dejnirattisai et al., 2021	IGHV1-58*01	IGHJ3*02	IGHD2-2*01		AAPACGTSCSDAFDI	IGKV3-20*01	IGKJ1*01		QQYGSSPWT
mAb165*^	Infection	(*42*)	IGHV1-58*01	IGHJ3*02	IGHD2-15*01		AAPHCIGGSCHDAFDI	IGKV3-20*01	IGKJ1*01		QQYGSSPWT

*not present in Figure 3C alignment

^Previously demonstrated to neutralize D614G

◊Previously demonstrated to neutralize D614G and viral variants B.1.17 and B.1.351 ((*16*); this study for 2C08)

Accessions: S2E12 PDB7K45;COV2-2196 MT763531.1 & MT763532.1; COV2-2381 MT66422.1 & MT665035.1; COV2-2072 MT665419.1 & MT665032.1; HbnC3t1p1_C6 MT658816.1 & MT658844.1; HbnC3t1p2_C6 MT658820.1 & MT658848.1; MnC5t2p1_G1 MT658833.1& MT658861.1
